# UVA and UVB Photoprotective Capabilities of Topical Formulations Containing Mycosporine-like Amino Acids (MAAs) through Different Biological Effective Protection Factors (BEPFs)

**DOI:** 10.3390/md17010055

**Published:** 2019-01-14

**Authors:** Francisca de la Coba, José Aguilera, Nathalie Korbee, María Victoria de Gálvez, Enrique Herrera-Ceballos, Félix Álvarez-Gómez, Félix L. Figueroa

**Affiliations:** 1 Department of Ecology and Geology, Faculty of Science, University of Malaga, Campus Universitario de Teatinos s/n, E-29071 Malaga, Spain; nkorbee@uma.es (N.K.); felix_alvarez000@hotmail.com (F.A.-G.); felix_lopez@uma.es (F.L.F.); 2 Photobiology Laboratory, Central Service for Research Support (SCAI), University of Malaga, Campus Universitario de Teatinos s/n, E-29071 Malaga, Spain; 3 Photobiological Dermatology Laboratory, Medical Research Centre, University of Malaga, Campus Universitario de Teatinos s/n, E-29071 Malaga, Spain; jaguilera@uma.es (J.A.); mga@uma.es (M.V.d.G.); eherrera@uma.es (E.H.-C.); 4 Department of Dermatology and Medicine, Faculty of Medicine, University of Malaga, Campus Universitario de Teatinos s/n, E-29071 Malaga, Spain

**Keywords:** Biological Effective Protection Factors (BEPFs), mycosporine-like amino acids, photoprotection, pH-thermo stability, UV- mediated action spectra

## Abstract

The safety and stability of synthetic UV-filters and the procedures for evaluating the photoprotective capability of commercial sunscreens are under continuous review. The influence of pH and temperature stressors on the stability of certain Mycosporine-like amino acids (MAAs) isolated at high purity levels was examined. MAAs were highly stable at room temperature during 24 h at pH 4.5–8.5. At 50 °C, MAAs showed instability at pH 10.5 while at 85 °C, progressive disappearances were observed for MAAs through the studied pH range. In alkaline conditions, their degradation was much faster. Mycosporine-serinol and porphyra-334 (+shinorine) were the most stable MAAs under the conditions tested. They were included in four cosmetically stable topical sunscreens, of which the Sun Protection Factor (SPF) and other Biological Effective Protection Factors (BEPFs) were calculated. The formulation containing these MAAs showed similar SPF and UVB-BEPFs values as those of the reference sunscreen, composed of synthetic UV absorbing filters in similar percentages, while UVA-BEPFs values were slightly lower. Current in vitro data strongly suggest that MAAs, as natural and safe UV-absorbing and antioxidant compounds, have high potential for protection against the diverse harmful effects of solar UV radiation. In addition, novel complementary in vitro tests for evaluation of commercial sunscreens efficacy are proposed.

## 1. Introduction

Terrestrial solar ultraviolet radiation (UVR) (295–400 nm) comprises UVA (320–400 nm) and UVB (295–320 nm). Although UVB accounts for no more than 5% of terrestrial UVR, their clinical effects on normal human skin are typically much greater than those of UVA. These effects, which are mostly adverse, may be acute or chronic. The most notable acute alterations caused by UVB radiation include erythema (sunburn), pigmentation (tanning), oedema, skinfold thickening, sunburn cells (SBC) formation, phototoxic reactions, photosensitivity and photoimmunosuppression [1]. Chronic effects include photocarcinogenesis and photoaging, also mediated by UVA [2]. All effects are underpinned by molecular or cellular effects such as DNA damage, generation of reactive oxygen species (ROS) (i.e., singlet oxygen, superoxide, peroxyl and hydroxyl radicals, peroxynitrite), melanogenesis, apoptosis, depletion of Langerhans cells, and expression of many genes and related proteins. On the other hand, the main widely-established benefit of UVR on the skin is the photosynthesis of vitamin D that is initiated by the UVB-induced conversion of epidermal 7-dehydrocholesterol into vitamin D3. Other positive effects are photo-adaptation (natural protection for the skin), immunosuppression of acquired immunity, induction of innate immunity, and recently identified, a reduction in blood pressure by UVA [3,4].

Recently, several effects of blue light (400–450 nm) on human skin have been reported, such as changes in pigmentation and erythema, thermal damage and free radical production [5,6]. In addition, near infrared radiation (A: 700–1400 nm) is associated with the production of ROS [7], resulting in collagen degradation and the appearance of coarse wrinkling, a characteristic of photoaging [8]. The application of high sun-protection factor (SPF) broad-spectrum sunscreens is considered an integral component of photoprotection for exposed skin [9]. 

The photobiological response to UVR depends on the energy associated with the incident photon flux and the relative effectiveness of photon energy to produce a given biological effect. This effectiveness is determined by the absorption spectrum of each skin chromophore (DNA, melanin etc.) which depends on its specific electronic configuration [10]. Action spectrum, a fundamental tool in medicine and photobiology, describes the relative effectiveness (used as a weighting function) of UVA and UVB wavelengths in the induction of health effects (i.e., DNA damage, skin cancer and erythema) usually expressed on a log scale.

Currently, the erythema (UVB-mediated skin injury) action spectrum is used for the in vitro SPF determinations, and Persistent Pigment Darkening (PPD) action spectrum for the UVA protection factor (UVA-PF). The European Union recommends both a critical wavelength of more than 370 nm and a UVA–PF of at least one third of the labelled SPF as the criterion for labelling as either UVA or broad-spectrum protection (ISO 24443:2012) [11]. However, other acute damages and most of the chronic ones are mediated by other wavelengths in the UV range, with known action spectra which could be used for complementary evaluations of sunscreen testing and labelling of broad-spectrum photoprotective capability in vitro. Especially, it may help to differentiate the effectiveness of sunscreen formulations with similar SPF in the future. Some of these action spectra are DNA damage [12], photocarcinogenesis non melanoma skin cancer (NMSC) [13], systemic immunosuppression of contact hypersensitivity (CHS) [14], cis-photoisomerization of urocanic acid [15], formation of oxygen radicals such as singlet oxygen [16] and photoaging [17], all related to UVB and UVA radiation exposure-induced skin damage.

The safety of sunscreen filters is being widely studied since there is evidence that synthetic organic filters are associated with allergic reactions or photo-toxicity [18,19,20], endocrine disruption [21], skin penetration [22], low photo-stability and biodegradability and a lack of effectiveness in skin protection [23,24]. Dibenzoilmethane, benzophenone, para-aminobenzoic acid (PABA) and their derivatives have been implicated as the cause of photoallergic dermatitis and the generation of oxygen radicals after solar exposition [25,26]. In addition, camphor derivatives showed skin permeation that permitted them to reach blood vessels located in deeper skin cell layers and thus entering the systemic circulation [27]. After long term exposure to UV, octocrylene produces ROS that accumulate in the body in measurable amounts, ultimately leading to cell mutations. Inorganic filters like TiO_2_ and ZnO offer broad spectrum protection but exposure to micro and nanoparticles of these filters could produce toxic effects [28]. In addition, commercial particulate UV filters, both inorganic and organic, are accumulating in coastal and continental waters [29,30], inducing a rapid and complete bleaching of hard corals even at extremely low concentrations [31]. UV filters have been found in invertebrates and fishes [32,33,34,35]. In addition, inorganic oxide nanoparticles containing UV filters such as TiO_2_ produce hydrogen peroxide, a major oxidizing agent entering coastal waters in touristic areas with direct ecological consequences to ecosystems [36].

There is a worldwide trend toward natural cosmetics and to the development of high UV protection sunscreens using low concentrations of chemical filters. The use of natural compounds in combination with synthetic agents may provide an effective strategy for preventing the harmful effects of UV radiation. Among the natural UV-absorbing molecules proposed in several studies as an alternative to synthetic filters, are flavonoids like quercetin and rutin (polyphenolic compounds) present in strawberries, grape fruits, apples, vegetables, tea, and red wine with multiple functions in photoprotection by suppressing the UV-induced damage in keratinocites [37]. In addition, some isoflavones like genistein and daidzein also block UVB induced skin burns in humans and show anti-photocarcinogenic and anti-photoaging effects [38]. Mycosporine-like amino acids have a good position since they are filters obtained from natural resources with no reported toxicity, high photo-stability and antioxidant properties [39,40,41,42,43]. Many studies suggest the use of MAAs as UV screen [44,45,46,47] and some extracts and formulations containing these compounds have been patented for the treatment of actinic erythema [48,49] as well as to avoid the oxidation of cosmetic and pharmaceutical products [50,51,52,53].

In this study, porphyra-334 (P-334), shinorine (SH) and asterina-330 (AS-330), with absorption maxima between 330–334 nm, mycosporine-serinol (M-Ser (OH)) with an absorption maximum at 310 nm and palythine (PNE) with an absorption maximum at 320 nm, were isolated in high purity grade and their thermo-stabilities at different pH and temperature conditions were evaluated. P-334 (+ SH), as a natural UVA sunscreen, and M-Ser (OH), as a natural UVB sunscreen, were brought together in the same topical formula as well as separately and SPFs and other Biological Effective Protection Factors (BEPFs) were calculated according to the Diffey method [48]. A reference sunscreen containing butylmethoxydibenzoylmethane (BMDM) and octylmethoxycinnamate (OMC), synthetic UV filters used in photoprotection by Cosmetics Europe [54,55,56], was formulated with the same percentage as that of the MAAs combination sunscreen and used as a control.

## 2. Results

### 2.1. Isolation and Purification of MAAs 

**Figure 1:**
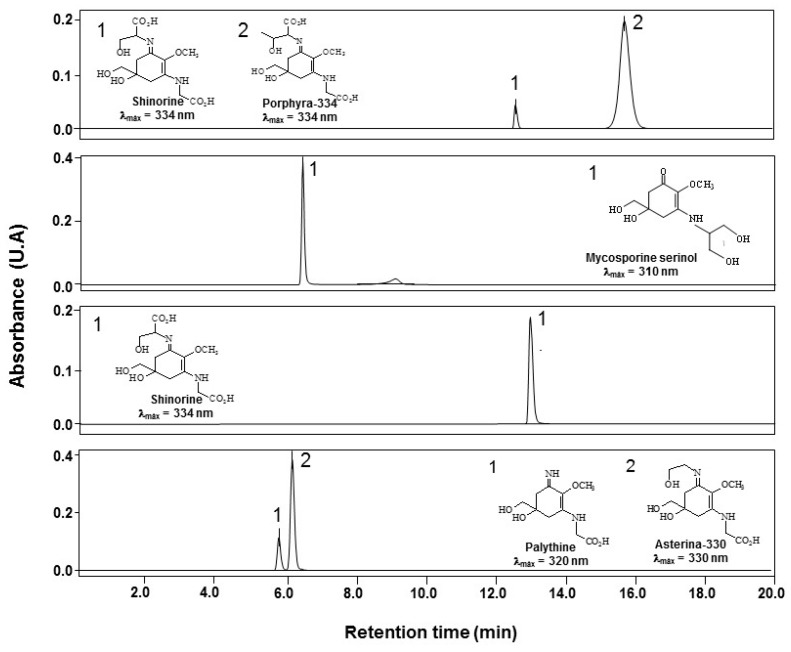
HPLC chromatograms of MAAs extracts after the Dowex chromatography purification process measured at the maximum absorption wavelength of the main MAA in each extract. The mobile phase was 1.5% aqueous methanol (*v*/*v*) plus 0.15% acetic acid (*v*/*v*) in water run isocratically at 0.5 mL min^−1^. Analyses were performed at 20 °C using a C8 chromatographic column.

Four different aqueous extracts containing MAAs with a high grade of purity were obtained. After HPLC and ESI-MS analysis, a combination of P-334 plus SH (88:12), AS-330 plus PNE (88:12), M-Ser (OH) plus an unknown UV absorbing compound with a similar absorption maximum (84:16), and SH as a unique MAA were identified and quantified (Figure 1). P-334 (+SH) and AS-330 (+PNE) results are expressed as percentages of each MAA with respect to total MAAs concentration, i.e., considering total MAAs the 100%. For M-Ser (OH) is referred to as the ratio of the chromatographic area of these two peaks. The ESI-MS analysis of purified MAAs showed a prominent ion peak of protonated molecules ([M + H]+) at *m*/*z* which is consistent with the MS analysis of MAAs derived from algae [57] (Table 1). Based on their on-line UV–visible absorption spectra and mass spectrometry, the MAAs were identified as porphyra-334 (UV λ_max_ 334 nm, *m/z* 347.1460) plus shinorine (UV λ_max_ 334 nm, *m/z* 333.130), mycosporine-serinol (UV λ_max_ 310 nm, *m/z* 262.1294), shinorine (UV λ_max_ 334 nm, *m/z* 333.130 and asterina-330 (UV λ_max_ 331 nm, *m/z* 289.1398) plus palythine (UV λ_max_ 320 nm, *m/z* 245.1135). In the case of the unknown molecule present in M-Ser (OH) extract, no concordance was found after checking 13 Mono-Substituted MAAs (Aminocyclohexenona-Type MAAs) with absorption maxima between 309-311 nm [58] , neither the selected glycosylated derivatives of porphyra-334 or shinorine in the extracts of P-334 (+SH) and SH.

### 2.2. Influence of pH and Temperature on MAAs Stability

**Figure 2:**
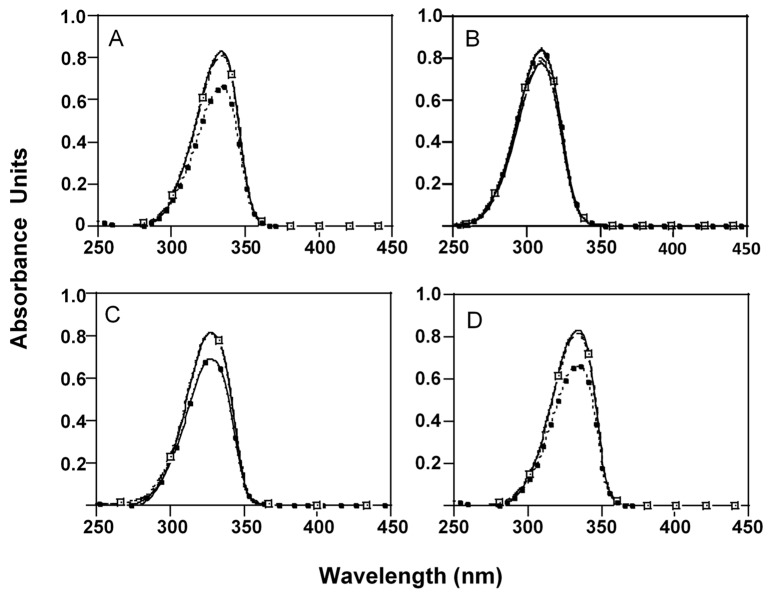
Absorption spectra of MAAs extracts after 24 h of incubation in 50 mM phosphate buffer pH 4 (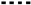), 7.5 (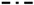), 8.5 (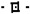) or 10.5 (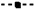) at room temperature (25 °C). Initial absorbance (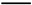). (**A**) P-334 (+SH), (**B**) M-Ser (OH), (**C**) AS-330 (+PNE) and (**D**) SH.

MAAs extracts incubated for 1 h at different pH conditions at room temperature (25 °C) did not show significant differences (*p >* 0.05) in concentration and absorption spectra. However, significant absorption decreases of MAAs (*p <* 0.05), except M-Ser (OH), were registered after 24 h of incubation at pH 10.5 (Figure 2). Initial absorbance of P-334 (+SH), SH, AS-330 (+PNE) decreased 18.4 ± 0.05%, 19.6 ± 0.04 and 13.8 ± 0.05 respectively.

**Figure 3:**
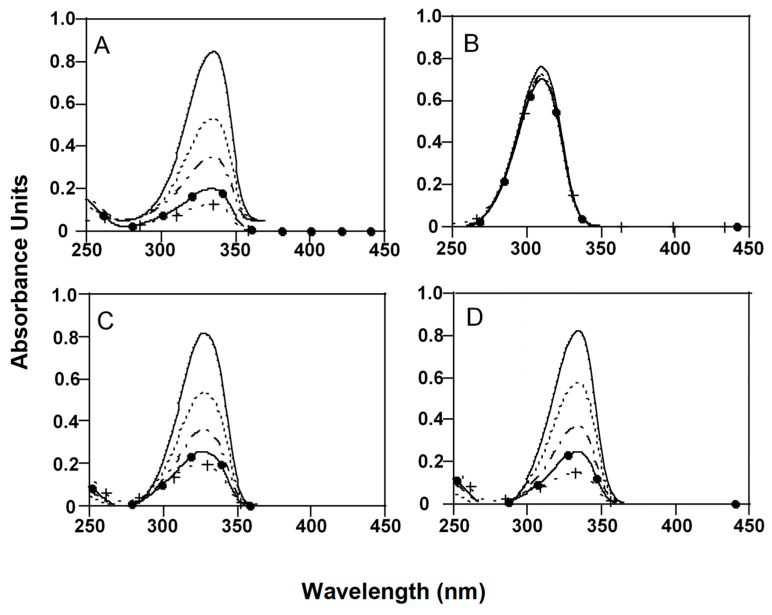
Absorption spectra of MAAs extracts after 1.5 (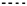), 3 (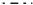), 4.5 (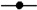) and 6 (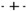) hours of incubation in 50 mM phosphate buffer at pH 10.5 and 50 °C. Initial absorbance (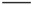). (**A**) P-334 (+SH), (**B**) M-Ser (OH), (**C**) AS-330 (+PNE) and (**D**) SH.

When the effects of pH and temperature on the stability of MAAs were analyzed, all MAAs were stable at 50 °C in buffer solutions with pH ≤ 8.5 (Figure 3) while their concentrations decreased in a time-dependent manner at pH 10.5, except M-Ser (OH), which remained stable (Figure 3B). After 1.5 h of incubation, AS-300 (+PNE) concentration decreased 33.3 ± 0.56%, SH 29.9 ± 0.63% and P-334 (+SH) 38.9 ± 0.45%. After 6 h, the concentrations decreased over 75% for all MAAs (Figure 3). In these conditions, UV-absorption maxima of AS-330 and P-334 (+SH) showed hypsochromic shifts from 330 nm to 334 and 334 to 333 nm respectively.

At 85 °C MAAs stability decreased with increasing the alkalinity of the medium (Table 2). In pH 4 solutions, P-334 (+SH), M-Ser (OH) and AS-330 (+PNE) registered decreases in absorbance of 17-18% (*p >* 0.05), while SH was smaller (*p <* 0.05). At pH 7.5, MAAs showed strong absorbance declines of up to 40% during the first 1.5 h of incubation while M-Ser (OH) was completely stable (*p >* 0.05). At pH 8.5, P-334 (+SH) and SH registered falls in absorbance of up to 90% after 1.5 h of incubation and their absorption maxima were 332 and 331 nm respectively. AS-330 (+PNE) resulted highly unstable, showing a single absorption peak at 304 nm unlike M-Ser (OH), the most stable MAA. All MAAs, except M-Ser (OH), were highly unstable at 85 °C and pH 10.5. Only small absorption peaks around 298–302 nm were measured during the first 1.5 h of incubation, indicating the presence of degraded products. M-Ser (OH) was also fairly unstable, but only after 3 h of incubation (Table 2).

In high basic solution (pH 10.5) and high temperatures (50–85 °C), both absorption maxima and extinction coefficients of the studied MAAs decreased in a time-dependent manner, registering the minimum values at 85 °C before 1.5 h of incubation for AS (+PNE) and after 3 h for M-Ser (OH). 

### 2.3. Galenic Formula

**Figure 4:**
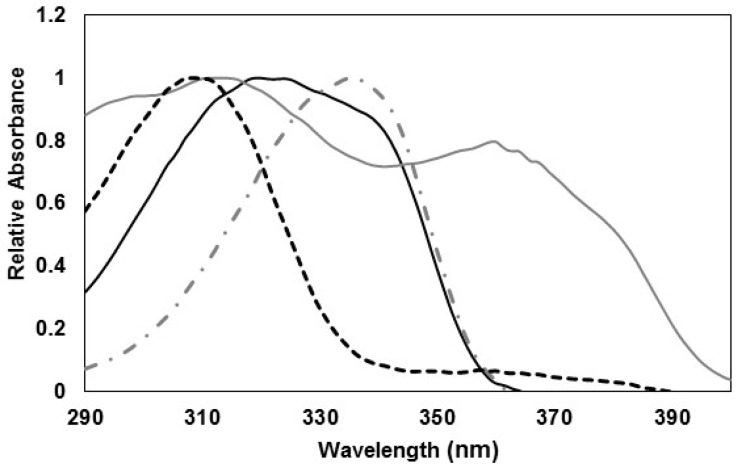
UV relative absorption spectra of different sunscreens studied: P-334 (+SH) plus M-Ser (OH) (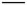) reference (OMC Y BMDM) (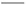), P-334 (+SH) (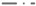) and M-Ser (OH) (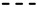).

After the preparation of topical emulsions, chemically stable and resistant to photo-degradation formulations were obtained. These creams were easily absorbed by the skin and able to create continuous and uniform films. The pH values of MAAs formulations were very homogeneous (6.2 ± 0.5). The sunscreens containing P-334 (+ SH) and M-Ser (OH) showed spectral absorption maxima at 334 (UVA) and 308 nm (UVB) respectively, corresponding to the absorption maxima of previous highly purified MAAs aqueous extracts (Figure 4). However, the absorption maximum of the sunscreen containing the mixture of these MAAs was 329 nm (UVA). The reference sunscreen, containing a combination of OMC and BMDM, showed two absorbance peaks at 310 and 360 nm, corresponding to λ maxima of each component (Figure 4).

### 2.4. SPF and BEPFs Determinations

SPF and BEPF values of the different sunscreens are presented in Table 3. The formulations containing P-334 (+SH) as a UVA absorbing filter or M-Ser (OH) as a UVB sunscreen presented similar SPFs (among 4-6) (*p >* 0.05), while the SPF of the formula containing the combination of these MAAs increased significantly up to 8.37 ± 2.12 (*p <* 0.05) and showed a similar values to those of the reference formula (9.54 ± 1.53). The λ critical values for P-334 (+SH), M-Ser (OH) and the MAAs combination sunscreens were 353.5 ± 3.0, 332 ± 1.5 and 343.6 ± 1.4 nm respectively. The reference were 374.4 ± 0.5 nm, being considered a broad-spectrum sunscreen.

In relation to UVB-BEPFs, P334 (+SH) sunscreen registered the lowest values in photoprotection against DNA damage, photocarcinogenesis (NMSC), and systemic immunosuppression. For the formation of singlet oxygen radicals and photoaging effects, M-Ser (OH) formula showed the lowest photoprotection. When BEPF values of separated MAAs (formulated at 5.6% for P-334 (+SH) and 5.1% for M-Ser (OH)) were compared with the MAAs combination emulsion (formulated at 4.5 and 2.6% respectively), the last one presented similar BEPF-UVB values to M-Ser (OH) (*p <* 0.05) and similar BEPF-UVA values to P-334 (+SH). Therefore, an additive effect was found when these MAAs were formulated together.

The MAAs combination sunscreen presented similar BEPF-UVB compared with the reference formula (*p >* 0.05). Photoprotection capabilities against the formation of singlet oxygen radicals and photoaging were significantly lower (*p >* 0.05).

## 3. Discussion

In recent years, mycosporine-like amino acids have gained considerable attention as highly active photoprotective candidates for the prevention of harmful effects of UVR on human skin, due to their physico-chemical characteristics. Many studies suggest the use of MAAs in topical creams [45,46,59]. High molar extinction coefficients (ε) or strong absorption in UVA and UVB regions allow to reduce the concentration of UV filters in sunscreens; ε values up to 20,000 M^−1^·cm^−1^ are considered effective for commercial sunscreens [60]. MAAs with molar extinction coefficients up to 50000 M^−1^ cm^−1^ are considered very effective UV filters, even more than other synthetic UVA absorber filters, such as BMDM (ε: 31000 M^−1^ cm^−1^). Their strong photo-stability in both aqueous solution [39,40,41,61] and seawater, as well as in the presence of abiotic stressors and strong photosensitizing agents and antioxidant properties, makes them considered safety sunscreens [42,52]. In fact, it was recently found that palythine showed only 3% degradation after an exposure of 40 standard erythema doses of solar simulated radiation [62]. However, the stability of these MAAs in a wide pH-temperature range have not been well studied, although it is very important to guarantee their stability both during and after they are added to cosmetic formulations.

Most synthetic UV filters, i.e., OMC and BMDM, present photo-stability problems. OMC degrades into photoproducts when it is exposed to sunlight for a short period of time, losing its efficiency in UV absorption [63]. BMDM has also been shown to be photo-labile after one hour of exposure to sunlight; this photo-decomposition not only reduces its photoprotective capacity by 50–60%, but also favors the generation of free radicals which causes ruptures in DNA plasmids and modifications in some proteins [64]. Some authors have even described the pro-oxidant nature of this filter in lipid peroxidation reactions [65]. The stability of both filters does not improve when they are formulated together, since irreversible photochemical reactions of cyclo-addition occur which lead to the loss of their photoprotective capacity [66]. To increase the stability of both filters, it is common to combine them with other substances (micronized ZnO/TiO_2_, salicylates, tinosorbs, etc.) [67,68]. 

In this study, we have demonstrated that P-334 (+SH), SH and AS-330 (+PNE) were very stable over a wide pH range (4.5–10.5) at room temperature in 24 hour experiments, registering slight absorbance decreases in alkaline solutions (pH 10.5), while M-Ser (OH) remained stable throughout all pH conditions. These results are in accordance with a previous work [69] in which porphyra-334 was found to be stable in solutions with pH from 1 to 11 up to 24 h at room temperature and rapidly degraded at extremely high pH 12 and 13. Mycosporine glycine, a MAA analogous to M-Ser (OH), showed high resistance to various pH conditions for up to 24 h [70,71]. The absorption maxima in the UV region of this MAA and shinorine were practically unaffected over the wide range of pH 4−10, being then the 3-aminocyclohexenone as well as the 1-amino-3-iminocyclohexene moieties, protonated at a wide range of pH, responsible for their UV-protective abilities in aqueous solution [72]. Combining high temperature and pH, MAAs absorbance decreased in a time-dependent manner and degradation products appeared; MAAs showed instability in alkaline buffer at 50 °C, and progressive disappearances were observed for MAAs through the studied pH range at 85 °C. In alkaline conditions, their degradation were much faster. 

The stability of porphyra-334 in solutions of different pH and temperatures was previously reported [73]. Only high acidic conditions, (pH below 3) and high alkaline conditions (pH over 12), as well as temperatures higher than 60 °C promotes its decomposition. A dehydrated sub-product of porphyra 334 with λ maxima at 226 nm was discovered at pH 7 and 120 °C [74]. Rastogi and Incharoensakdi [75] showed very high stability of partially purified extracts of palythine and asterina-330 from a cyanobacterium at 60 °C after 1 hour assay. In our study, degraded products of P-334 (+SH) and AS-330 (+PNE) were only detected at 50 °C and pH 10.5 after 6 h of incubation, in accordance with Rastogi and Incharoensakdi [75] and Korbee et al. [76]. MAAs showed the minimum concentration values at 85 °C and pH 4-10.5 before 1.5 h of incubation for AS-330 (+PNE) and after 3 h for M-Ser (OH). 

Among the pH-temperature conditions studied, M-Ser (OH) and P-334 (+SH) were found to be the most stable MAAs and asterina-330 the most labile one. According to these results, M-Ser (OH) was selected as natural UVB filter and P-334 (+SH) as natural UVA filter to be added to different topical galenic emulsions and evaluating their photoprotective capabilities in vitro. Different MAAs sunscreens were obtained according with the safety pH-temperature range defined in this work: pH 6 and 60 °C.

Although the need to prevent acute (erythema) and chronic skin damage (cancer and photoaging) resulting from exposure to ultraviolet radiation (UVB and UVA) is well understood, the safest and most effective way to achieve this protection still presents a number of challenges, specifically in the practical implementation of photoprotective measures. SPF only provides information regarding the photoprotective capacity of topical sunscreen products against the erythematic action of ultraviolet radiation. It does not provide data regarding the level of protection against other UV- mediated biological effects. In this context, this study proposes a novel method for the evaluation of the in vitro photoprotection capacity of topical formulations for the main UV-induced skin injuries (acute and chronic disorders) using their action spectra and transmission measurements. Thus, the photoprotection capacity of the studied formulations based on MAAs purified extracts has been established for a total of 7 UV-mediated biological effects with action spectra referenced in the Photobiology literature (Table 4). BEPF can be understood as the number of times a person can be exposed to the sun compared to an unprotected person before this person suffers the biological injury in question.

In this study, we demonstrated that two galenic emulsions containing purified MAAs (P-334 (+SH) or M-Ser (OH)) in similar percentages as natural UVA and UVB filters (5.6 or 5.1%, respectively) showed similar SPF values, providing similar photoprotection against erythema. The extinction molar coefficient of P-334 (+SH) is much higher than M-Ser (OH) in spite of its UVA maximum absorption. Both MAAs sunscreens registered λ critical values below 370 nm, indicating they are not broad-spectrum sunscreens. According to these results, both MAAs could be used indiscriminately as sunscreens against erythema, with P334 (+SH) being the best candidate to provide even greater protection against UVA (λ critical higher). When P334 (+SH) and M-Ser (OH) were included in lower concentrations in the same formula, the SPF of this sunscreen was significantly similar to the SPF of M-Ser (OH), denoting a possible synergistic effect between them due to both broadband covering of UV solar spectrum and high extinction molar coefficients. When these results were compared with the reference sunscreen which contains the same percentage of UVA+UVB synthetic filters, we found similar SPF for both formulas. These results confirm that these MAAs provide great photoprotection in vitro against erythema and their potentiality for possible substitutions of synthetic UV filters. From an economic point of view and regarding our experience with these compounds, a high quantity of fresh material is necessary in order to get the required amount of purified MAAs. Purification yields of 40–50% were obtained for the MAAs studied. For this reason, the manufacturing of sunscreens with P-334 (+SH) as unique UV filter with high SPF (30–50) is not easy to achieve as large amounts of biomass would be necessary. Furthermore, due to the regulation of the individual filters in sunscreen formulas, in this case, the combination of different filters would be necessary. MAAs could be good candidates to be used together with other commercial filters. In the case of cosmetics with 100% natural filters, commercial viability could be reached with a low SPF formula (as hydration creams).

There is a lot of research that supports the relationship between the photoprotective role against UVA and UVB harmful diseases of porphyra334 (and shinorine) in vitro and in vivo experiments. Our research group [78] studied the in vivo UV-protective properties of purified porphyra-334 and shinorine isolated from *P. rosengurttii.* Results showed that the galenic formulation containing P-334 (+SH) included at 2% (w/w) with SPF 3.71 and critical wavelength 357–358 nm, applied topically at a concentration of 4 mg· cm^-2^ on the dorsal skin of SkhR-1 H hairless mice and irradiated with a single UV radiation dose of 3.87 J·cm^−2^ (3.6 MEDs for a human phototype of skin III/5.1 MEDs for a phototype IV) prevented UV-induced clinical and histopathological skin alterations observed in non-UV-protected biopsies. The expression of the heat shock protein Hsp70, a potential biomarker of acute UV damage, and the antioxidant defense enzymes also were preserved. In addition, Porphyra-334 protected cell viability in vitro studies with keratinocytes (human HaCat) and reduced the DNA damage in fibroblasts (IMR-90) and dimers formation [79,80]. Regarding UVA-mediated injuries, Porphyra-334 suppressed ROS production and the expression of matrix metalloproteinase following UVA irradiation, while increasing levels of procollagen, type I collagen and elastin [81,82]. 

Erythema is the end point of the main index of sunscreen efficacy, SPF, which is mainly, but not exclusively, an index of UVB protection. The time-point for evaluating erythema and the erythematic doses for each human phototype of skin are fully standardized parameters, but for most of the biological effects shown in Table 4, these circumstances do not occur, and some of them begin to develop at sub-erythematic doses [83]. Additionally, high-SPF products tend to lull users into staying in the sun longer and overexposing themselves to both UVA and UVB rays. In view of this perspective, to provide more useful information to consumers, more studies are necessary to study the biologically effective doses for each biological effect in order to find safe UV-exposure limits that may be equivalent to minutes of sun exposure (immediate or long-term photobiological and degenerative skin changes). Nevertheless, high UVA-BEPF values for a galenic sunscreen formula are correlated to high UVA range protection as UVB-BEPF values to high UVB protection in this study. 

When the photoprotective capability of these different sunscreens were analyzed against other UV-mediated injuries, M-Ser (OH) showed higher UVB-BEPFs values than P-334 (+SH), although their SPF were similar, unlike for UVA-BEPFs. Although the formation of singlet oxygen radicals and photoaging process are both mediated mainly by UVA radiation (96%) and both present the maxima effectiveness at 340 nm, the solar effective irradiance between 350-380 nm is higher for the photoaging response than formation of singlet oxygen. Therefore the protective capability of P-334 (+SH) is higher for this injury. The combination of MAAS sunscreen showed similar UVB-BEPFs to those of M-Ser (OH) and the reference formula, indicating an additive photoprotective effect between P-334 (+SH) and M-Ser (OH) for these UVB injuries and a similar effectiveness to that of synthetic UV filters. The combination of MAAs sunscreen (λ critical 343.6 nm) presented similar UVA-BEPFs values to P-334 (+SH) one (λ critical 353.5 nm) and very close to the reference formula (λ critical 374.4) nm. However, these formulas could be enriched with other natural UVA-absorbing substances like scytonemin (λ_max_ 384 nm) to increase λ critical rate and then, broad spectrum absorption. 

Therefore it is demonstrated that topical sunscreens containing a combination of MAAs P-334 (+SH) and M-Ser (OH) provide excellent photo-protective in vitro capabilities against the main UV-mediated injuries compared to chemical UV filters. For UVB mediated process, an additive photoprotection effect is observed when these MAAs are used together, unlike many associations of synthetic UV filters used in commercial sunscreens. This effect is smaller for UVA injuries. Currently, BEPFs indicators could be useful tools to compare the UVA+UVB protection given by different commercial sunscreen products beyond their SPF and λ critical values.

Due to a greater understanding of the effects of visible light and infrared radiation, it is now clear that available organic (chemical) UV filters are not sufficient to protect the skin. Only optically opaque filters such as non-micronized form of zinc oxide, titanium dioxide and iron oxide are able to block visible light, but these galenic forms are unacceptable to many users [84]. In addition, photoprotection long after UV exposure is being demanded since the generation of DNA damage and the synthesis of cyclobutane pyrimidine dimers (CPDs) are believed to occur hours after UV exposure. The so-called dark CPDs are mediated by ROS and the use of systemic or topical antioxidants could prevent their synthesis [85]. Natural compounds with broad biological activities are important in the manufacturing of sunscreen products due to their modulation of several signaling pathways in reducing the deleterious effects of UV radiation on the skin. Antioxidants topically applied and their oral and subcutaneous topical forms of photoprotection are ongoing and the protective effect of β-carotene topically applied [86] and *Polypodium leucotomos* extracts [87] have been proven. In addition, the antioxidant properties of MAAs have been widely studied [58,71,88], specifically the purified MAAs tested in this study, in terms of scavenging hydrosoluble radicals (ABTS assay), inhibition of β-carotene oxidation, and superoxide radical scavenging [42] describing moderate activity of P-334 (+SH) and high activity of Mycosporine glycine (analogous to M-Ser (OH)) and AS-330 (+PNE) relative to ascorbic acid and vitamin E.

## 4. Materials and Methods

### 4.1. Isolation, Identification and Characterization of MAAs 

Three red macroalgae (*Porphyra rosengurttii*, *Gelidium corneum* and *Ahnfeltiopsis devoniensis*) and one marine lichen (*Lichina pygmaea*) were selected for this study, considering their high contents in P-334 (4.85 ± 0.31 mg/g DW), AS-330+PNE (0.57 ± 0.03 mg/g DW), SH (0.8 ± 0.17 mg/g DW) and M-Ser (OH) (1.1 ± 0.23 mg/g DW) respectively. The extraction and isolation of these MAAs in high purification grade was carried out by adsorption and ionic exchange chromatography based on published protocols [59,89] modified by De la Coba et al. [42]. MAAs were identified both by HPLC according to Korbee-Peinado et al. [90] and by using Electrospray Ionization Mass Spectrometry (ESI-MS). For HPLC analysis, a Waters 600 HPLC System (Waters Cromatografía S.L., Barcelona, Spain) was used with a mobile phase of 1.5% aqueous methanol (*v*/*v*) plus 0.15% acetic acid (*v*/*v*) in water, run isocratically at 0.5 mL min^−1^. Sample volumes of 10 μl (μg/mL MAAs concentrations) were injected into a C8 chromatographic column (4 μm average particle size, 250 × 4.6 mm; Luna, Phenomenex, Aschaffenburg, Germany) with a guard column (C8, Octyl, MOS, Phenomenex). Autosample temperature was maintained at 20 °C. MAAs were detected online with a Waters Photodiode Array Detector 996 (Waters Cromatografía S.L., Barcelona, Spain), and absorption spectra (290–400 nm) were recorded each second directly on the HPLC-separated peaks. Mycosporine-like amino acids were also analyzed by mass spectrometry (ESI-MS) with a high-resolution mass spectrometer (model Orbitrap Q-Exactive, Thermo Scientific, Bremen, Germany) provided with an electrospray ionization-heated probe (HESI-II), in the premises of the Central Service for Research Support (SCAI, University of Málaga, Málaga, Spain). This technique ionizes molecules while preserving their structures, unlike low resolution techniques that involves the breakdown of analytes. The identification of MAAs was according to comparing their protonated theoretical molecular masses *m/z* [M + H]^+^ with the parent ions detected, with an allowed approximation of 4 decimals. The samples were dissolved in 100% methanol in order to μg/L MAAs concentrations. Mass calibration for Orbitrap was performed once a week, in both positive and negative modes, to ensure a working mass accuracy lower than or equal to 5 ppm. The HESI parameters were optimized as follows: sheath gas flow rate 12 arbitrary unit; auxiliary gas flow rate 0 arbitrary unit; capillary temperature 320 °C; heater temperature 50 °C; spray voltage 4.0 KV and S lens RF level 50. Full scan data in positive polarity mode were acquired at a resolving power of 140,000 FWHM (full width half maximum) at *m/z* 200, and a scan range of *m/z* 100–700 was chosen. The AGC (Automatic gain control) target was set to 10^6^, with a maximum injection time of 30 ms. 

MAAs were identified firstly by HPLC by comparing absorption spectra, retention times and by co-chromatography using high purified grade MAAs extracts provided from the Unit of Photobiology of the Central Service for Research Support (SCAI, University of Málaga, Málaga, Spain). Their molecular structures were confirmed by High- resolution Electrospray Ionization Mass Spectrometry (ESI-MS) using their protonated molecular masses with an accuracy to the fourth decimal. Molecular structures of glycosylated derivatives of porphyra 334 (478–508 Da) and shinorine (464 Da) described by Nazifi et al. [91] with absorption maxima at 334–335 nm were also checked in ESI-MS spectra of P-334 (+SH) and SH extracts. Quantification was carried out using published extinction coefficients [89,92,93,94,95] extracting chromatographic peak areas of each MAA at its maximum absorption wavelength. 

### 4.2. pH MAAs Stability Determination

The different aqueous purified extracts were diluted in a final volume of 1 ml phosphate buffer (50 mM) at different pH conditions (4.5, 7.5, 8.5 and 10.5) with an initial absorbance of 0.8 ± 0.05 in each MAA solution. Absorbance spectra (from 250 to 450 nm) were measured after 1 and 24 h of incubation using a UV-Vis spectrophotometer Shimadzu 1603 (Shimadzu Co., Kyoto, Japan). All determinations were carried out in triplicate at room temperature (25 °C). 

### 4.3. Temperature and pH MAAs Stability Determination

In order to evaluate the combined effect to different temperature and pH conditions, purified MAA extracts were incubated at 50 °C and 85 °C during 6 h using screw cap tubes to avoid volume loss. After that time, in a final volume of 1 ml, the different aqueous purified extracts were diluted in phosphate buffer (50 mM) at different pH values (4.5, 7.5, 8.5 and 10.5) to an initial absorbance of 0.8 ± 0.05 in each MAA solution. Absorbance spectra (from 250 to 450 nm) were measured 1.5, 3, 4.5 and 6 h post-incubation. All determinations were carried out in triplicate. 

### 4.4. Galenic Formulations

In order to analyze the MAAs photoprotection capabilities in vitro, different oil/water emulsions were formulated containing a mixture of Neo PCL self-emulsifying base (Acofarma, Barcelona, Spain) and propylene glycol in water as the base formula. This formulation presented pH 5.8. Different aqueous filters were added to the propylene glycol-water phase prior to mixing with Neo PCL. The ratio of these three components was 4:1:14 (expressed in *w*/*v* for Neo PCL and *v*/*v* for propylene glycol and water extracts) and 19.6% *w*/*v*, 4.9% *v*/*v* and 68.6% *v*/*v* in the final formula respectively. The galenic compositions were formulated at 60 °C (the safety range for MAAs according to this study). New pH measurements of MAAs sunscreens were taken once they were tempered. Absorbance spectra were measured at a rate of 2 mg · cm^−2^ of the different sunscreens on 3M Transpore self-standing substrates. The products were distributed uniformly in a circular motion with a finger equipped with a rubber thimble for about 30 s. The preparations were allowed to dry for 15 minutes and then were irradiated using a double band UV spectrophotometer Shimadzu UV-1800 (Shimadzu Co., Kyoto, Japan) between 290–400 nm wavelengths. Transpore substrate with base formula was used as a baseline.

Four emulsions were formulated: P- 334 (+ SH) at 5.6% (w/w); M-Ser (OH) at 5.1% (w/w); a combination of these MAAs where P-334 (+ SH) were the majority MAAs (4.1 vs. 2.9 %); and finally, a reference cream containing a combination of UVA and UVB-absorbing reference filters: BMDM, UVA1 filter (λ_max_ between 355–359 nm) (Fagrón Ibérica S.A., Barcelona, Spain) and OMC, UVB filter (λ_max_ 311 nm) (Fagrón Ibérica S.A., Barcelona, Spain) with similar rate to the MAAs combination one (4.5 and 2.6% respectively). 

### 4.5. In vitro Sun Protection Factor (SPF) Determination

SPF was characterized according to the Diffey method [96] with some modifications, and protection in the UVA band was calculated by the critical wavelength [97,98]. The UVA/UVB ratio defines the performance of a sunscreen in the UVA range (320–400 nm) in relation to its performance in the UVB range (290–320 nm), while the critical wavelength is given as the upper limit of the spectral range from 290 nm on, within which 90% of the area under the extinction curve of the whole UV range between 290 and 400 nm is covered. When the critical wavelength is 370 nm or greater, the product is considered broad spectrum, which denotes balanced protection throughout the UVB and UVA ranges.

Transmittance measurements were performed on 3M surgical Transpore self-standing substrate, a material chosen to simulate the roughness of human skin with better correlation between in vitro and in vivo SPF measurements when the test is not pre-irradiated [99]. Transpore tape was stuck onto a light-diffusing double- ground quartz plate and placed on an analytical balance. The sunscreens were plotted onto the tape from a pre-weighted syringe at a rate of 2 mg·cm^−2^ along the selected surface (4 cm^2^) and spread evenly with a gloved finger for about 30 s. The preparation was allowed to dry for 15 min and then, was irradiated using a double band UV spectrophotometer (Shimadzu UV-1800) between 290–400 nm wavelengths, collecting the resulting transmittance spectrum. A Transpore substrate with base formula was used as a baseline.

The sun protection factor (SPF) was calculated from transmission measurements according to:

$$\mathrm{SPF}=\sum_{290}^{400}E(\lambda )\cdot S\;(\lambda )/\sum_{290}^{400}E(\lambda )\cdot S\;\left(\lambda \right)\cdot T\;\left(\lambda \right)$$

where

Eλ = CIE standard Skin reference erythema action spectrum [77].

Tλ = Transmitance values (0–1).

Sλ = Spectral irradiance of a midday clear sky of summer, terrestrial sunlight in Spain (22 June at 36.71° N, −4.47° W).

Sunlight was measured by using a portable double monochromator spectrorradiometer Bentham IDR300-PSL (Bentham Co., Reading, UK) which takes measurements in W m^−2^·s^−1^ between 200 to 500 nm (Device located at Unit of Photobiology of the Central Service for Research Support, SCAI, University of Malaga, Malaga, Spain). 

### 4.6. Biological Effective Protection Factors (BEPFs)

BEPF calculation for a determined UV-mediated skin response was obtained through transmission measurements according to the Diffey method using the relative action spectrum for this biological effect (Table 4). BEPF can be understood as an indicator given to sunscreen consumers, informing them about the number of times a photoprotected person can be exposed versus a person with unprotected skin before this process begins to develop.

**Figure 5:**
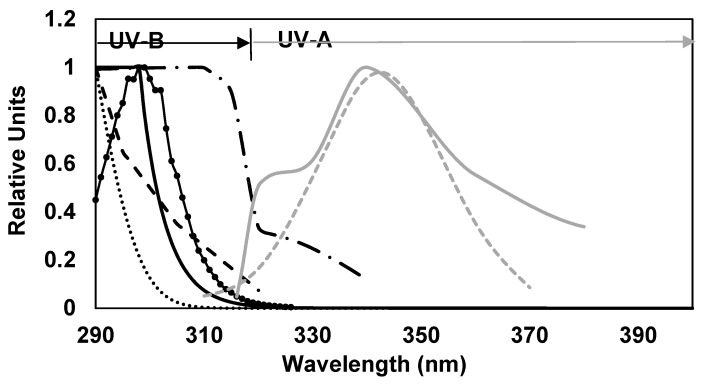
Normalized Action Spectra (290–400 nm) for different UVB and UVA mediated harmful biological effects used in this study: DNA Damage (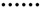), erythema (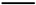), photocarcinogenesis (NMSC) (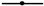), induction of systemic suppression of CHS (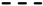), photoisomerization of urocanic acid (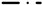), formation of singlet oxygen (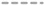) and photoaging (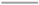).

In this study, the level of UV photoprotection for the different formulated emulsions was determined for a total of seven UV-biological mediated processes with a well-known action spectra associated with human skin. Some of them have maximum effect on UVB (UVB-BEPFs) and others in the UVA wavelengths (UVA-BEPFs) (Table 4). Data for erythema, DNA damage, and photocarcinogenesis of non-melanoma skin cancers (NMSC- SCUP-h) spectra were available in the literature [12,13,14]. For the other action spectra selected, cubic splinic interpolation between the data points of the respective action spectrum has been employed to provide values of 1 nm increments and the integral in the Equation (1) replaced by a summation in 1 nm step. Splinic interpolation was done by means of the software Table curve 2D 5.0.1. (Systat Software Inc., San Jose, CA, USA). The error in the interpolation and summation in 1 nm step is estimated to be lower than 5% (Figure 5).

### 4.7. Statistical Analysis

The results are presented as the mean value ± standard deviation of at least 4 samples. The statistical significance of the means was contrasted by one-factor Analysis of Variance (ANOVA) followed by Tukey’s multiple comparison test [100]. A fixed level of significance of 95% (*p* < 0.05) was used in all cases.

## 5. Conclusions

The high stability of MAAs over a wide range of temperature and pH, together with their antioxidant properties in aqueous and lipid solutions and photoprotective capabilities against adverse effects of UVB and UVA solar radiation (patented), make them excellent cosmeceutical compounds, which have grown exponentially over the years and have become feasible substitutes of active products used in some medical treatments. The discovery of new active agents, such as those of marine origin, is currently the focus of intense research. We have also proposed new complementary in vitro tests to evaluate the efficacy of commercial sunscreens that we recommend to be included in the information given to consumers.

## 6. Patents

Part of the results obtained in this research were registered in the patent “López-Figueroa, F.; Aguilera Arjona, J.; de la Coba-Luque, F.; Korbee Peinado, N. Composición para protección solar a base de extractos de algas y líquenes. ES Patent 2,317,741, A1, 16 April 2009”.

## Figures and Tables

**Table 1:** ESI-MS results of MAA extracts in high grade of purity.

Fraction	MAAs	Mol.formula	UV λ_max_ (nm)	Exact (ppm)	*m/z* [M + H]^+^	
					Parent Ion	Theoretical
**P-334 (+SH)**	**Porphyra-334**	C_14_H_22_N_2_O_8_	334	3.1	347.1460	347.1449
	**Shinorine**	C_13_H_20_N_2_O_8_	334	2.5	333.1301	333.1292
**M-Ser (OH)**	**Mycosporine-serinol**	C_11_H_19_NO_6_	310	3.4	262.1294	262.1285
**SH**	**Shinorine**	C_13_H_20_N_2_O_8_	334	3.0	333.1302	333.1292
**AS-330 (+PNE)**	**Asterina-330**	C_12_H_20_N_2_O_6_	331	1.3	289.1398	289.1394
	**Palythine**	C_10_H_16_N_2_O_5_	320	1.3	245.1135	245.1132

**Table 2:** Absorbance decreases (%) of different MAAs at 85 °C and different pH conditions for 4.5 h of incubation. The values are expressed as mean ± SD (*n* = 4). Different letters indicate significant differences (*p* < 0.05) between values for each MAAs and pH condition.

MAAs Extract	pH	Time (Hours)		
		1.5	3	4.5
**P-334 (+SH)**	4	6.9 ± 0.04 ^a^	11.2 ± 0.04 ^b^	18.3 ± 0.05 ^c^
	7.5	41.8 ± 1.19 ^a^	67.6 ± 1.62 ^b^	80.4 ±1.12 ^c^
	8.5	93.5 ± 0.07 ^a^	**100** ^b^	
	10.5	**100**		
**M-Ser (OH)**	4	7.4 ±1.03 ^a^	14.7± 1.92 ^b^	17.4 ± 2.03 ^b^
	7.5	-	6.4 ± 0.08 ^a^	10.0 ± 0.08 ^b^
	8.5	17.2 ± 0.03 ^a^	20.7 ±0.04 ^b^	25.1 ± 0.04 ^c^
	10.5	69.5± 0.06 ^a^	**100** ^b^	
**AS-330 (+PNE)**	4	-	-	12.6 ± 0.05
	7.5	54.9 ± 1.23 ^a^	77.6 ± 1.56 ^b^	86.7 ± 0.08 ^c^
	8.5	**100**		
	10.5	**100**		
**SH**	4	-	12.8 ± 0.03 ^a^	17.6 ± 0.03 ^b^
	7.5	50.6 ± 2.03 ^a^	74.6 ± 1.87 ^b^	85.4 ± 1.23 ^c^
	8.5	89.8 ± 0.08 ^a^	**100** ^b^	-
	10.5	**100**		

**Table 3:** SPF and BEPFs values for tested formulations : P-334 (+SH) at 5.6%, M-Ser (OH) at 5.1%, P-334 +(SH) at 4.1% plus M-Ser (OH) at 2.9% and the reference containing BMDM at 4.5% and OMC at 2.6% (w/w). The values are expressed as mean ± SD (*n* = 4). Different letters indicate significant differences (*p <* 0.05) between different purified MAAs extracts for a UV-mediated effect (BEPF).

UV-mediated Effects	P-334 (+SH)	M-Ser (OH)	MAA Combination	Reference
**Erythema (SPF)**	4.53 ± 1.58 ^a^	6.47 ± 1 ^ab^	8.37 ± 2.12 ^bc^	9.54 ± 1.53 ^c^
**DNA Damage**	4.17 ± 1.55 ^a^	9.27 ± 1.98 ^b^	10.18 ± 2.99 ^b^	9.71 ± 1.58 ^b^
**Photocarcinogenesis (NMSC)**	4.60 ± 1.63 ^a^	7.55 ± 1.31 ^b^	8.74 ± 2.24 ^b^	9.50 ± 1.49 ^b^
**Systemic Immunosuppression (CHS)**	5.63 ± 2.23 ^a^	9.73 ± 2.17 ^b^	10.72 ± 2.99 ^b^	10.41 ± 1.68 ^b^
**Urocanic acid photoisomerization**	7.22 ± 3.21 ^a^	9.21 ± 2.37 ^a^	10.90 ± 2.95 ^a^	11.00 ± 2.38 ^a^
**Formation of singlet oxygen radicals**	6.51 ± 2.25 ^b^	2.62 ± 0.18 ^a^	6.45 ± 1.69 ^b^	9.74 ± 1.89 ^c^
**Photoaging**	4.81 ± 1.72 ^b^	2.16 ± 0.10 ^a^	4.37 ± 0.94 ^b^	10.63 ± 2.63 ^c^

**Table 4:** Absorption maxima, UVB/UVA relative effectiveness (%) and experimental model for the selected UV-mediated biological injuries.

Biological Effects	λ_max_ (nm)	UVB/UVA	Experimental Model
**Erythema**	250–298	99/1	Human skin [77]
**DNA damage**	270	100/0	Function [12]
**Photocarcinogenesis (NMSC)**	298	99/1	SCUP-h (human) [13]
**Inmunosuppression CHS**	270	100/0	Balb/c mouse [14]
**Photoisomerization of urocanic acid**	303–309	86/14	Human skin [15]
**Formation of Singlet oxygen**	342–343	4/96	In vitro [16]
**Photo-aging**	340	4/96	SKHR-1 H mouse [17]
